# Effects of a lumbar exoskeleton that provides two traction forces on spinal loading and muscles

**DOI:** 10.3389/fbioe.2025.1530034

**Published:** 2025-03-21

**Authors:** Kaicheng Qi, Zhiyang Yin, Chao Li, Jianjun Zhang, Jingke Song

**Affiliations:** ^1^ School of Mechanical Engineering, Hebei University of Technology, Tianjin, China; ^2^ Hebei Provincial Key Laboratory of Robot Sensing and Human-Machine Integration, Tianjin, China; ^3^ Engineering Research Center of the Ministry of Education for Intelligent Rehabilitation Devices and Testing Technology, Tianjin, China

**Keywords:** lumbar exoskeleton, traction forces, intervertebral disc pressure, stiffness optimization, electromyography

## Abstract

In workplaces with prolonged or repetitive stooping, disc herniation due to excessive pressure at the lumbar L5/S1 joints has been a difficult condition to prevent and treat in the field of lower back pain. Previous research on lumbar exoskeletons mainly focused on the impact of assistive torque on muscle activation, overlooking the fact that providing assistive torque is not the optimal approach when bending over with a low load. Instead, using traction force to reduce disc pressure is a more adaptable method to mitigate the risk of intervertebral disc herniation. In this paper, a novel lumbar exoskeleton mechanism is proposed. The exoskeleton principle is similar to a lever arranged on the torso, which provides two types of traction forces using a single compression spring with a lower support moment and higher traction performance. Subsequently, a kinetic simulation model covering passive physiologic tissues and spring stiffness was developed to determine the optimal range of traction forces for a given load, to predict the disc pressure and muscle activation at optimal traction forces. Eight subjects were invited to wear the exoskeleton for stooping and lifting tests under extreme loads, using myoelectric sensors to measure muscle activation. The results confirm that optimal traction force effectively reduces L5/S1 disc pressure without additionally increasing muscle activation. The exoskeleton in this study provides an alternative idea for the design of lumbar exoskeletons adapted to light load stooping.

## 1 Introduction

Lower back pain has been a significant factor in worker productivity in fields such as agriculture, manual labor, medical care, and manufacturing (Friedrich et al., 2000; Keawduangdee et al., 2012; D’ERRICO et al., 2013; Fethke et al., 2015). To reduce the risk of workers suffering from lower back pain, a range of active and passive exoskeletons have been developed and put into production (De Looze et al., 2016; Ali et al., 2021; Kranenborg et al., 2023).Active exoskeletons use motors as a drive source to provide sufficient power for the human, but active exoskeletons are structurally heavy and have a short range, workers are more likely to accept passive exoskeletons (Govaerts et al., 2024; Poliero et al., 2022).Currently, exoskeletons tend to assist stooping by generating extension moments. If the moment curve is idealized, it can make the back muscles not to exert any force at all. Laevo, as a classic mass-produced industrial exoskeleton (Moya-Esteban et al., 2023; Luger et al., 2023; Hwang et al., 2021),is simple in structure and easy to wear. The exoskeleton is fitted with a cam-spring booster at the hip joint, which generates a supportive extension moment when the worker is stooping. The exoskeleton achieves a compact design that significantly reduces the size of the rigid booster mechanism, however, due to the size constraints of the cams, the exoskeleton requires an extremely high spring stiffness to generate an effective torque. The SPEXOR developed by Näf MB(Koopman et al., 2020b; Näf et al., 2018) has two elastic energy storage modules to generate torque, but the exoskeleton provides too much torque, which is more favorable in lifting situations above 15 kg. For low load bending, the excessive torque will severely interfere with the kinematics of the wearer’s natural bending state, making it necessary for the user to change the movement strategy. Some flexible exoskeletons, such as the biomechanical garments represented by PLAD (Lamers et al., 2017; Abdoli-e and Stevenson, 2008; Abdoli-Eramaki et al., 2007; Frost et al., 2009), use stretchable bands to simulate the back muscles generating force along the tendons. Since the bands have a longer force arm, these exoskeletons have an advantage in reducing the activation of the lower back muscles.

Previous exoskeleton assistance mechanisms primarily aimed to augment human muscle force, with electromyography (EMG) testing often serving as a standard for evaluating exoskeleton performance (Moya-Esteban et al., 2023; Abdoli-E et al., 2006). However, spinal load is a crucial indicator of lumbar injury risk, and changes in kinematics or reductions in muscle force do not necessarily correspond to a decrease in spinal loads (Picchiotti et al., 2019; Madinei and Nussbaum, 2023; Koopman et al., 2020a). In reality, the body undergoes a “flexion-relaxation phenomenon” during stooping, a natural strategy to reduce energy expenditure. At a certain degree of lumbar flexion, passive tissues such as ligaments contribute the majority of the extension moments, leading to a “silent” phase in the active electromyographic signals of the erector spinae muscles (McGill and Kippers, 1994; Toussaint et al., 1995; Colloca and Hinrichs, 2005; Solomonow et al., 2003). At this stage, the body requires little external assisting moment and may be more sensitive to the moment demands of the exoskeleton. Therefore, the moment demands on the lumbar exoskeleton are not as great at this time. This situation is more likely to occur in low load stooping manual work such as agriculture or handicrafts rather than large object handling tasks. In that case, following a conventional exoskeleton would not be conducive to reducing L5/S1 spinal loading (Eskandari et al., 2025). A blanket reduction of active electrical signals from the muscles may not be optimal. In contrast, due to the shorter moment arm of the lumbar ligaments, there remains a risk of spinal loads (Toussaint et al., 1995). Excessive spinal load, which may lead to intervertebral disc injury, is a critical factor in evaluating lower back pain (Jaffar Nur and Abdol, 2017), yet it is often overlooked in exoskeleton design. Traction force can increase disc height, providing a mechanical effect that alleviates spinal pressure and mitigates disc herniation (Zaïri et al., 2021; Tadano et al., 2019; He et al., 2024; Chow et al., 2017; Pellecchia, 1994). However, exoskeletons capable of delivering tractional support are still yet to be developed. Chaerim Moon (Moon et al., 2022) proposed a lower back exoskeleton with a 4-bar linkage structure, using a tensile spring with a stiffness of 11,700 N/m to generate traction and extension torque. The system employs a tension sensor to estimate a traction force of approximately 100 N.

This paper makes the following contributions: 1) We propose a novel back exoskeleton that, unlike traditional designs which primarily generate moments to assist human motion, takes into account the flexion-relaxation phenomenon observed in lumbar passive tissues during flexion-extension movements. This exoskeleton respects the contributions of passive muscle force and ligaments in deep flexion. The exoskeleton mechanism is designed to enhance traction force at the expense of some extension moments. Theoretically, this conversion allows the exoskeleton to avoid impeding human movement, with the reduced extension moments minimizing the risk of abnormal abdominal muscle activation. From a biomechanical perspective, this exoskeleton is more adaptable to work environments involving frequent lifting tasks with light or zero loads. 2) We developed a dynamic simulation framework for the interaction between the human and the back exoskeleton. This framework identifies the optimal exoskeleton stiffness under 8 kg-load conditions, analyzing its combined effects on muscle coordination, spinal load, and muscle activation. The simulation is constrained to sagittal plane two-dimensional motion and passive exoskeleton mechanics (assuming all exoskeleton structures are lightweight, without affecting traction mechanics). Previous motion models often predicted movement trajectories as solutions to optimization problems, which could result in lumbar motion angles that do not accurately reflect natural human movement characteristics. In this study, we focus on how traction force in the back exoskeleton influences muscles and intervertebral discs during standard flexion-extension movements, with inverse dynamics optimization serving as the main direction of our research.

## 2 Exoskeleton design

### 2.1 Design overview

**FIGURE 1:**
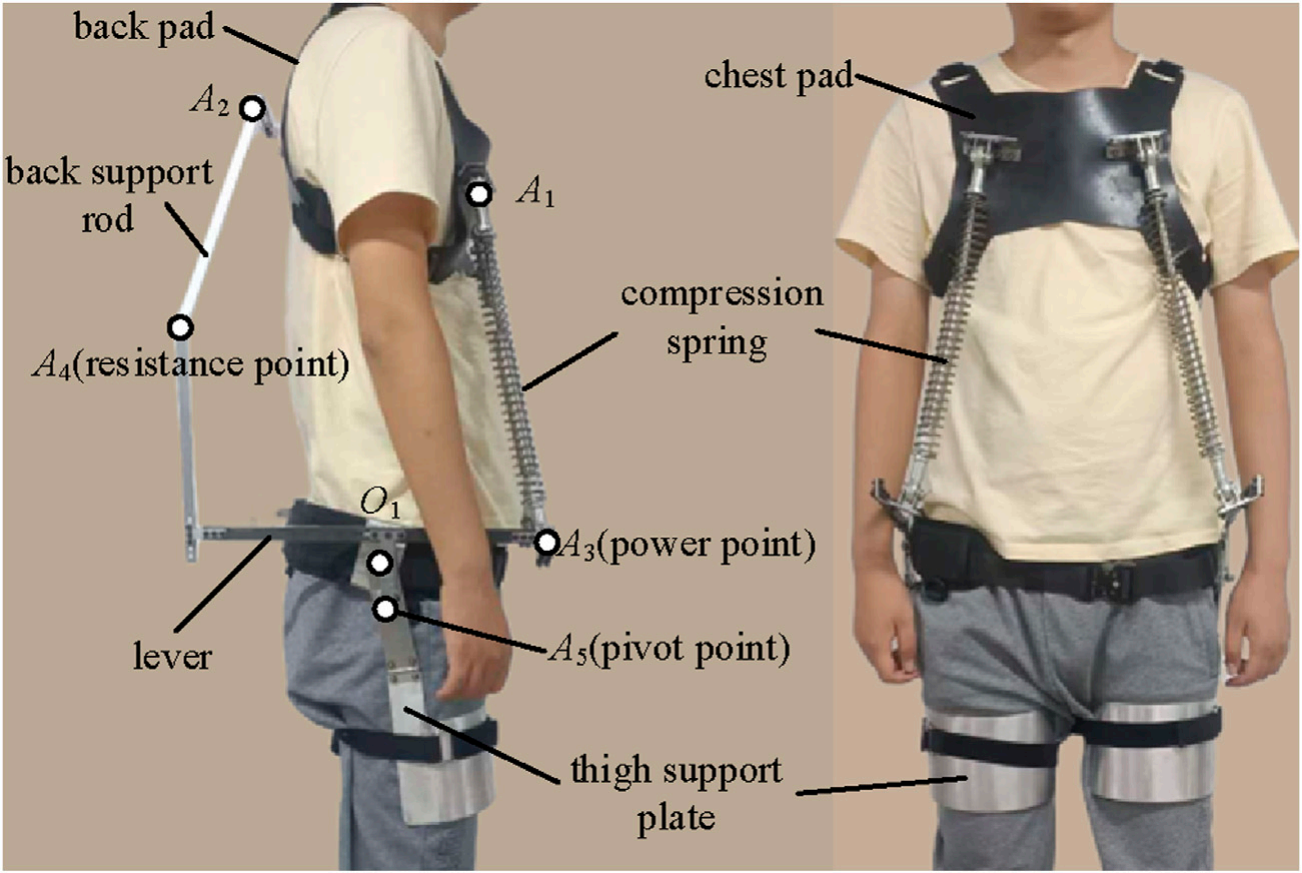
Scheme configuration of the exoskeleton side view. *A*_4_ *A*_5_ *A*_3_ are connected to form a lever rod, with *A*_3_ as the power point, *A*_4_ as the resistance point, and *A*_5_ as the lever pivot point. Chest traction force is directed along *A*_3_ *A*_1_ and back traction force is directed along *A*_4_ *A*_2_.

The back exoskeleton we designed has the same structure on the left and right sides of the body (Figure 1), with the overall structure symmetrically arranged along the sagittal plane. The exoskeleton mechanism resembles a lever placed on each side of the body. When the body bends forward, the compression spring gradually compresses, generating two equal but opposite spring forces. The upward spring force is applied to the chest pad, pulling the upper body upward; we refer to this as the chest traction force. The downward spring force is distributed at the *A*_3_ point, acting as the driving force at the power point of the exoskeleton lever. The resistance force is distributed at the lever’s resistance point *A*_4_ on the back. If the back support rod is considered a two-force bar, an equal and opposite force is generated at the back pad position, directed upward along the back support rod; we call this the back traction force. The connection joint between the lever and the thigh support plate serves as the pivot point *A*_5_, allowing the lever to reach a balanced state through the interactions of these elements. The reaction force at the pivot is transferred to the thigh support plate.

### 2.2 Traction force model

**FIGURE 2:**
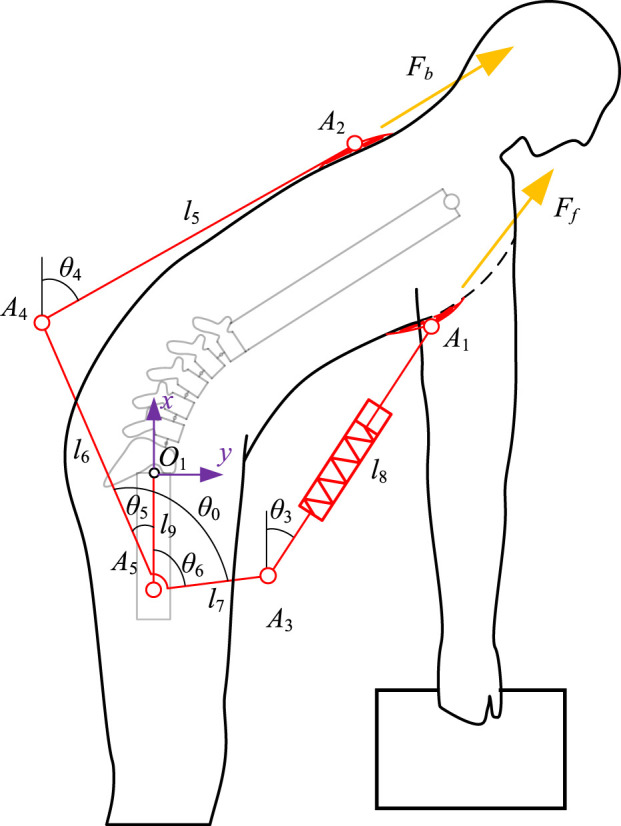
Sketch of the mechanism consisting of a human and an exoskeleton. The sketch simplifies the spinal part of the human body, defines the angles and dimensions of the exoskeleton used to solve the kinematics and traction forces, and labels the direction of the chest traction force *F*_*f*_ and the back traction force *F*_*b*_.

**FIGURE 3:**
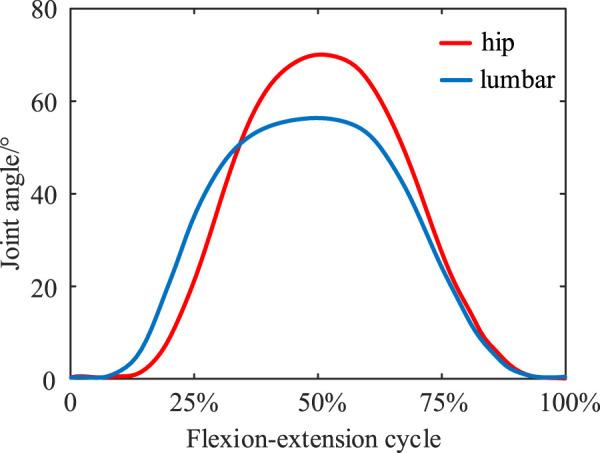
Synergistic movement patterns of the lumbar spine joints and hip joints during standard flexion and extension exercises.

A simplified sketch of the human-exoskeleton mechanism (Figure 2) allows to analyze the relationship between the upper body motion and the compression of the spring, with reference to the angles of the lumbar spine and hip joints under standard flexion-extension movements measured by Pal et al. (2007) (Figure 3), synergistic movement patterns of the vertebrae by Wong et al. (2006), to calculate the traction law generated by the exoskeleton under a standard flexion-extension cycle.

An absolute coordinate system is established at the hip joint *O*_1_ (note: all angles are referenced to this coordinate system), then two closed-loop vector equations are created to describe the mechanism motion (Equations 1, 2):

$$\left\{\begin{array}{l} x_{A_{2}}=l_{6}\cos\theta_{5}+l_{5}\cos\theta_{4}-l_{9}\\ y_{A_{2}}=l_{6}\sin\theta_{5}+l_{5}\sin\theta_{4} \end{array}\right.$$ (1)

$$\left\{\begin{array}{l} x_{A_{1}}=l_{7}\cos\theta_{6}+l_{8}\cos\theta_{3}-l_{9}\\ y_{A_{1}}=l_{7}\sin\theta_{6}+l_{8}\sin\theta_{3} \end{array}\right.$$ (2)

where (*x*_*A*2_, *y*_*A*2_) and (*x*_*A*1_, *y*_*A*1_) are the coordinates of point *A*_2_, position of the exoskeleton tied to the back, and point *A*_1_, position of the exoskeleton tied to the chest, respectively, in the absolute coordinate system. To simplify the model, the left and right limbs are assumed to be perfectly symmetrical during flexion and extension movements, so we model the human body as a multi-rigid linkage in the sagittal plane (Manns et al., 2017). Due to the limited range of independent motion of the head and thoracic spine relative to the lumbar spine (Consmüller et al., 2012), we treat them as a single rigid body. The lumbar region is represented as a sequentially articulated mechanism with five vertebrae and the pelvis connected by revolute joints. The elbow and wrist joint angles are fixed, and the arms are assumed to remain vertical throughout the flexion-extension process to simulate the natural hanging posture of the arms during bending and lifting. Since flexion primarily requires small joint angles at the knees and ankles (Bazrgari et al., 2007; Carnegie et al., 2022), the entire lower limb is treated as a rigid body fixed to the ground. Subsequent analyses will disregard the kinematic influence of the lower limbs on the human body and exoskeleton. The whole human body model consists of 8 rigid bodies including arms, thoracic vertebrae, lumbar vertebrae, sacrum, and thighs, with 6 degrees of freedom (lighter lines in Figure 2).

The geometric dimensions and inertia parameters of the vertebrae are based on measurements by Kuo et al. (2010), Pearsall et al. (1996). The coordinates of the points *A*_2_, *A*_1_ with the movement of the upper body were obtained using the homogeneous coordinate transformation method, after which the variation of the spring length could be calculated (Equations 3, 4):

$$\left\{\begin{array}{l} l_{8}=\sqrt{{\left(x_{A_{1}}+l_{9}-l_{7}\cos\theta_{6}\right)}^{2}+{\left(y_{A_{1}}-l_{7}\sin\theta_{6}\right)}^{2}}\\ \theta_{3}=\frac{y_{A_{1}}-l_{7}\sin\theta_{6}}{x_{A_{1}}+l_{9}-l_{7}\cos\theta_{6}} \end{array}\right.$$ (3)

$$\left\{\begin{array}{l} \theta_{5}=\arcsin\frac{h_{0}\sin\varphi }{h_{1}}-\arccos\frac{h_{1}^{2}+l_{6}^{2}-l_{5}^{2}}{2h_{1}l_{6}}\\ \theta_{4}=\arcsin\frac{h_{0}\sin\varphi -l_{6}\sin\theta_{5}}{l_{5}}\\ \varphi =\arctan\frac{y_{A_{2}}}{x_{A_{2}}}\\ h_{0}=\sqrt{x_{A_{2}}^{2}+l_{9}^{2}+y_{A_{2}}^{2}}\\ \begin{array}{l} h_{1}=\sqrt{h_{0}^{2}+l_{9}^{2}+2h_{0}l_{9}\cos\varphi }\\ \theta_{6}=\theta_{0}+\theta_{5} \end{array} \end{array}\right.$$ (4)

Consider an ideal compression spring that, with damping and preload force neglected, produces a chest traction force (spring force) of (Equation 5):

$$F_{f}=k_{s}\left(l_{s0}-l_{8}\right)$$ (5)

where *k*_*s*_ is the sum of the spring stiffnesses of the exoskeleton on both sides and *l*_*s*0_ is the initial length of the spring of the human body in the standing phase. Assuming that the exoskeleton consists entirely of lightweight rods, we can derive the static equilibrium equations for the lever. This allows us to determine the mathematical relationship between the two types of traction forces (Equation 6):

$$F_{b}=\frac{F_{f}l_{7}\sin\left(\theta_{6}-\theta_{3}\right)}{l_{6}\sin\left(\theta_{5}+\theta_{4}\right)}$$ (6)

where *F*_*b*_ is the back traction force. The mathematical model predicts the trend of the two traction forces generated by the exoskeleton under standard flexion and extension movements.

The moments for the body due to chest and back traction are calculated by the cross-multiplication of the vectors from points *A*_1_ and *A*_2_ to the L5/S1 joints and the vectors of the chest and back traction forces, and the sum of the two moments is the total moment of the exoskeleton.

### 2.3 Stiffness optimization

According to the traction force model, there is a direct relationship between exoskeleton spring stiffness and traction force. The amount of traction force affects the body’s muscle force generation. Too little traction force can lead to insufficient assistive effect, and the disc pressure will remain at a high threshold, which is not effective in avoiding spinal injuries. Too much traction force may lead to negative muscle activity, which in turn may cause injury to the human body. By establishing a musculoskeletal-based kinetic model, the effect of traction force on the musculoskeletal system under the phenomenon of flexion relaxation can be simulated, and the decompression performance of the exoskeleton with optimal traction force can be obtained.

**FIGURE 4:**
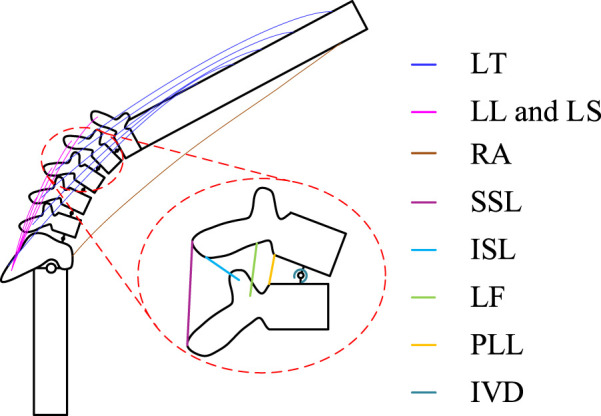
Schematic diagram of the tissues that make a major contribution during flexion-extension movements. It contains the distribution of the erector spinae muscle groups, the distribution of lumbar spine ligaments.

Due to the complexity and large number of low back muscle groups, we simplify the calculations by modeling only the erector spinae muscle as the main force-generating muscle group in flexion and extension, with a total of 22 muscle fibers. Within this muscle group, muscle fiber bundles are divided into the thoracic longissimus (LT), lumbar iliocostalis (LL), and lumbar longissimus (LS) based on the different attachment points of the muscle fibers (Figure 4). In contrast, the rectus abdominis (RA) was also modeled to represent the antagonist muscle of the longest muscle of the thoracic spine. Muscle contraction dynamics are described using the hill model (Millard et al., 2013), expressed as follows (Equation 7):

$$F_{m}=F_{om}\left(a_{m}F_{am}+F_{pm}\right)$$ (7)

where, *F*_*m*_ is the total muscle force, *F*_*am*_ is the dominant force factor, *F*_*pm*_ is the passive force factor, *F*_*om*_ is the maximum isometric force of the muscle fiber, *a*_*m*_ is the muscle fiber activation.

The posterior longitudinal ligament (PLL), ligamentum flavum (LF), supraspinous ligament (SSL), and interspinous ligament (ISL), which play a primary role in the flexion-extension movement, were selected (Figure 4). The attachment points of these four lumbar spine ligaments on the vertebrae were determined according to the method described by Naserkhaki et al. (2018). The ligament stiffness data followed the force-deflection curves determined by Pintar et al. (1992) for ligaments of the lumbar T12-L2, L2-L4, and L4-Sl segments. For computational convenience, nonlinear stiffness curves for all ligaments were fitted with a sixth-degree polynomial, which when multiplied by the ligament stretch can be expressed as the ligament force *F*_*l*_. The intervertebral disc joint was modeled as a torsion spring with constant stiffness in the coronal axis to calculate the disc’s resistance moment *M*_*v*_, and their stiffness data were obtained from (Senteler et al., 2016).

The Newton-Euler method was employed to establish the multi-body dynamics equations for each rigid body (Equation 8), which can visualize the contact forces between different vertebrae, as well as the contributions made by active and passive muscle forces, ligaments, and intervertebral discs to the generation of extension moments. This, in turn, facilitates the assessment of intervertebral disc pressures. The L5/S1 intervertebral disc pressure, which is a combination of disc moments, muscle forces, ligament forces, and vertebral joint forces *F*_*x*_, *F*_*y*_, can be written as:

$$F_{c5}=F\left(F_{m},F_{l},F_{x},F_{y},M_{v}\right)$$ (8)

**FIGURE 5:**
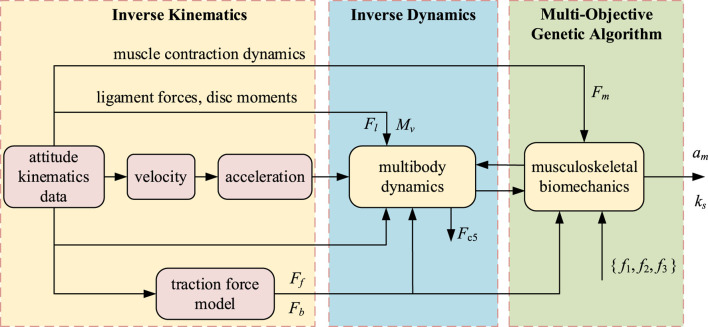
Simulation flow of spring stiffness and muscle activation level, an inverse dynamics calculation method. The simulation results show the optimized spring stiffness, the degree of muscle activation before and after the traction force is applied and the L5/S1 disc pressure.

Since the unknown amount of muscle fiber activation is much larger than the number of equations in the kinetic model, optimization algorithms are required to obtain solutions, while different objective functions lead to variability in the optimization results (Ostraich and Riemer, 2024). We formulate the muscle as well as the stiffness solution problem as a multi-objective optimization problem (Figure 5) with constraints based on the dynamical equations of Newton’s Euler method and a range of muscle activation *a*_*m*_. Considering that with a spring stiffness of 2000 N/m the exoskeleton traction force will be close to the effective traction force threshold of 500 N (Zaïri et al., 2021), the stiffness does not exceed this value. It is constructed as follows (Equations 9, 10):

$$\min\;f\left(a_{m},k_{s}\right)=\left\{f_{1},f_{2},f_{3}\right\}$$ (9)

$$s.t.\;\begin{array}{l} \left\{\begin{array}{c} ma=\sum F_{x}+\sum F_{y}+\sum F_{m}+\sum F_{l}\\ J\ddot{\theta }=\sum F_{x}\times l_{x}+\sum F_{y}\times l_{y}+\sum F_{m}\times l_{m}+\sum F_{l}\times l_{l}+\sum M_{v} \end{array}\right\}\\ 0\leq a_{m}\leq 1 \end{array}$$ (10)

where *m* represents the mass, *J* represents the rotational inertia, *a* represents the acceleration, and $\ddot{\theta }$ represents the angular acceleration of the joint. *l*_*x*_, *l*_*y*_, *l*_*m*_, *l*_*l*_ are the force arms of each component of the force relative to the disc rotating the joint, respectively.

The first objective function is the minimization of the sum of squares of muscle activation (Equation 11), which means that the sum of work done by human muscles is minimized. It is widely applied in various muscle optimization solution problems (Rahmati et al., 2017; De Groote et al., 2016; Manns et al., 2017). Unlike other methods, the optimization values of muscle activation in this function are related to the corresponding maximal isometric force, indicating that muscle fibers with greater force capacity will tend to exert more force.

$$f_{1}=\sum_{i=1}^{22}\frac{200{\left(a_{m,i}\right)}^{2}}{F_{om,i}}$$ (11)

The second objective function employs the total sum of squared differences to minimize the variation in activation levels among all muscle fibers (Equation 12). This reflects the body’s ability to neurally marshal muscle, with similarity in activation changes of muscle fibers that play the same role when muscle groups are stimulated through the same or approximate reflex pathway.

$$f_{2}=\sum_{i=1}^{22}\sum_{j=i+1}^{22}{\left(a_{m,i}-a_{m,j}\right)}^{2}$$ (12)

The third objective minimizes the cumulative reaction force and moment at the L5/S1 intervertebral disc over a flexion-extension cycle (Equation 13), with *w*_1_, *w*_2_ and *w*_3_ representing weight coefficients, *F*_*L*5*/S*1_, *M*_*L*5*/S*1_, and *M*_*hip*_ representing the L5/S1 force, the L5/S1 moment, and the hip moment, respectively. This reflects the body’s tendency to generate active muscle forces in a way that minimizes skeletal load (Brown and Potvin, 2005). Although the optimization principles of these three objective functions may conflict, they together capture the true activation patterns of the erector spinae muscles during bending and lifting movements. Including exoskeleton spring stiffness in the optimization framework does not shift the optimization direction; on the contrary, it represents an optimal stiffness that reduces spinal load and muscle activation without compromising muscle coordination.

$$f_{3}=w_{1}\left\|F_{L5/S1}\right\|+w_{2}\left\|M_{L5/S1}\right\|+w_{3}\left\|M_{hip}\right\|$$ (13)

The simulation framework was constructed and executed in MATLAB 2022b, with optimization conducted using a multi-objective genetic algorithm (MOGA) (Deb, 1999). MOGA balances multiple objective functions by finding a set of non-dominated solutions (Pareto optimal solutions) to achieve the best compromise between different objectives. The L5/S1 intervertebral disc pressure before wearing the exoskeleton was solved by assigning the degree of muscle activation through a multi-objective genetic algorithm, so as to assign a reasonable muscle force to each joint to satisfy the equilibrium of kinetic states. The spring stiffness was optimized according to the peak pressure of L5/S1 joints, and the L5/S1 disc pressure after wearing the exoskeleton was optimized by incorporating the traction force. The simulation timestep was set to 0.1 s, with the model starting in an initial upright posture, bending to the lowest point, then returning to an upright stance, covering a total duration of 4 s. This setup allows the simulation to capture real-time loading on intervertebral discs and changes in activation levels across individual muscle fibers of the erector spinae. For ease of analysis, the muscle fibers were grouped into three major muscle groups—thoracic longissimus, lumbar iliocostalis and longissimus, and rectus abdominis. The activation level of each muscle group was represented by the sum of activations of the fibers within that group.

## 3 Experiment

### 3.1 Subjects and procedures

A total of eight healthy male subjects were invited to participate voluntarily in the trial, with a mean age of 24 ± 1.5 years, mean height of 176 ± 8.3 cm, and mean weight of 73.3 ± 5.8 kg. All subjects had no report of low back pain in the last 3 months and had not been involved in large lifting tasks. Before the experiment, all subjects read the instructions and precautions related to the experiment and signed an informed consent form for the experiment. The experiment was approved by the Ethical Review Committee of Hebei University of Technology.

Raw EMG data were recorded using a Noraxon Ultium Wireless Surface EMG Tester (Noraxon Inc., United States) with a sampling frequency of 2000 Hz. The skin of the subjects was shaved before the experiment, and then cleaned of grease and other contaminants by wiping with an alcohol cotton ball. The surface EMG sensors were attached to the thoracic erector spinae (TES), lumbar erector spinae (LES), and rectus abdominis (RA), of which the thoracic erector spinae and lumbar erector spinae were mainly responsible for trunk flexion and extension, and the rectus abdominis muscle was responsible for trunk flexion. Before the experiment, the sensor light was covered with black tape to prevent the motion capture system from misjudging it as a Marker point. The kinematic data of the joints were recorded using a Vicon T40-S Motion Capture System (Oxford Metrics Ltd., United Kingdom) with a sampling frequency of 200 Hz. Reflex point markers were attached to T7, T12, S1, PSIS, ASIS, hip, mid-thigh, knee, mid-calf, ankle, and tip of the foot. The angle of the vector formed between the labeled points was used as the angle of joint movement during the experiment.

### 3.2 Procedures

The experiment was divided into four groups: No weight in hands without exoskeleton, 8 kg weight in hands without exoskeleton, no weight in hands with exoskeleton, and 8 kg weight in hands with exoskeleton. During the complete experiment, subjects were asked to keep their arms parallel to the direction of gravity at all times. Without the exoskeleton, the subjects were bending from the initial standing position in a natural descending motion to the subjective conscious limit, and then returned to the standing position in a natural rising motion without artificial intervention of synergistic movements between lumbar vertebrae and hip joints. There is no artificial interference with the synergistic movement between the lumbar spine and the hip joints. The phases from standing to bending, and from rising to standing, were all approximately 2 s. The choice of 0 kg was made in view of the fact that even without a load, the disc pressure during bending is not optimistic, and back pain in farmers has been reported in the field of agricultural harvesting or cultivation (Fathallah, 2010). 8 kg was chosen because it is the load level at which the NOISH lifting criteria for L5/S1 disc compression can reach the injury risk threshold (McGill and Kippers, 1994).The two loads represent most of the typical light-duty load-bearing work situations. Subjects wore the exoskeleton and started the experiment with a bending and rising motion without the exoskeleton as the norm, causing a supervisor to observe the subject’s posture in real time and to remind and correct his movements, using a metronome to control the tempo of the task. Subjects were made to not bend their knees and ankles as much as possible. If the knee and ankle flexion angles exceeded a certain threshold, the data from this set of experiments were nullified. This is because if the knee and ankle joints were overflexed it would affect the phenomenon of flexion relaxation (Shin et al., 2004),and would not be conducive to the observation of the connection between the exoskeleton and the EMG signals. All experimental situations were repeated six times, and after completing one set of experiments, the subjects were allowed to take a full rest for 10 min to prevent muscle fatigue.

### 3.3 Data processing

The original EMG signals were first imported into MATLAB, and the raw data were filtered using a fourth order Butterworth bandpass filter. Afterwards, the data were subjected to full-wave rectification and the RMS values of the EMG data were plotted. To ensure the comparability of electromyography (EMG) data among different subjects, the EMG signal data were normalized. Prior to the experimental tasks, the maximum voluntary contraction (MVC) of each subject was measured using the manual resistance method on a Roman chair (Biviá-Roig et al., 2019). The experimental signal values of each group were divided by the MVC signal values to obtain the normalized data, expressed as MVC%. This normalization allows signals from different individuals or different experimental conditions to be adjusted to the same scale, enabling direct comparisons between signals from different datasets or conditions and facilitating subsequent processing and analysis steps. The Kolmogorov - Smirnov test was employed to examine the normality of the electromyography (EMG) signal parameters. Subsequently, a paired t - test (α = 0.05) was utilized to evaluate whether there were significant differences in the peak values of the MVC% signals before and after wearing the exoskeleton.

## 4 Results

### 4.1 Effects of traction force on spinal loading

**FIGURE 6:**
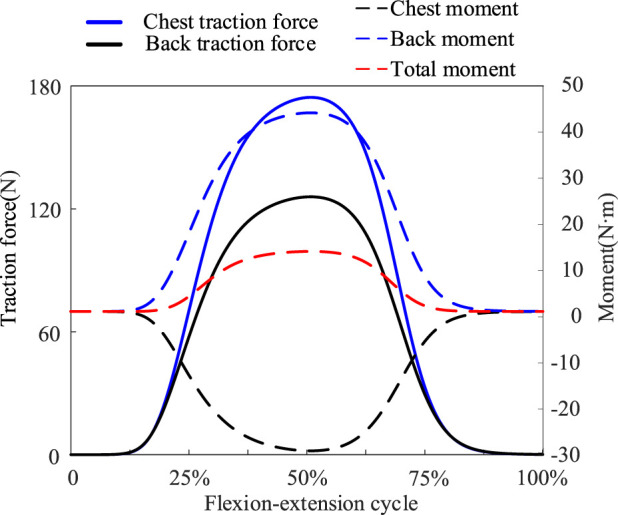
Based on the traction mechanics model, chest traction and back traction were calculated. Chest traction force produces an extension moment, back traction force produces a flexion moment, and a portion of both moments cancel out, creating a lower total extension moment provided by the exoskeleton.

**FIGURE 7:**
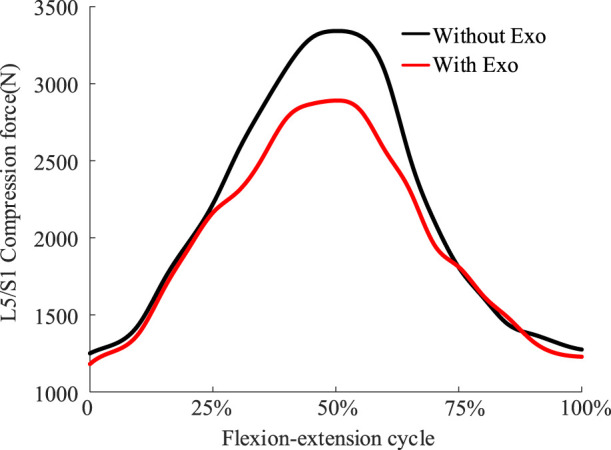
Comparison of disc compression forces at the L5/S1 joints before and after wearing the exoskeleton under an extreme load of 8 kg.

Simulation results showed that the total stiffness of the optimized spring under 8 kg load was 1233 N/m. The chest and back traction provided by the exoskeleton increased with increasing flexion (Figure 6). The exoskeleton provided almost no traction and moment at the 0%–15% and 85%–100% stages. This is because at this stage the lumbar joints are less than 22.1°, the hip joints are less than 14.2°, and the angle of inclination of the human trunk is small enough that no more traction or extension moments are required. The exoskeleton provided maximum traction when the body reached 50% of the final stage of flexion, with thoracic traction of 173.4 N and dorsal traction of 125.5 N. The flexion moment generated by the dorsal traction partially offset the extension moment generated by the thoracic traction, making the overall extension moment generated by the exoskeleton appear not to be excessive (Figure 6). At the point where the body reaches 50% of the final stage of flexion, the maximum extension moment is just 13.1 N m. Lower support torque and higher traction performance may be better adapted in workplaces oriented to bending up under light loads or 0 kg loads (Zhang et al., 2023). Multiple traction forces demonstrated an excellent mechanical effect of reducing disc pressure (Figure 7), with the main reduction reflected in the peak phase of disc pressure, where peak compression forces were reduced from 3,326.4 N to 2,873.2 N.

### 4.2 Effects on the erector spinae muscle

**FIGURE 8:**
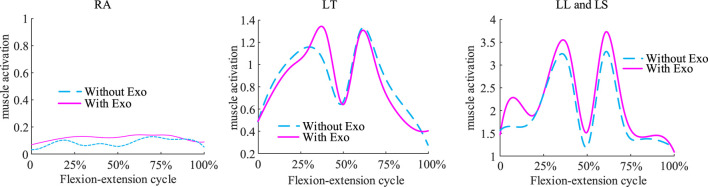
Comparison of muscle activation before and after wearing the exoskeleton under an extreme load of 8 kg. Muscle activation had a similar trend with or without wearing the exoskeleton.

**FIGURE 9:**
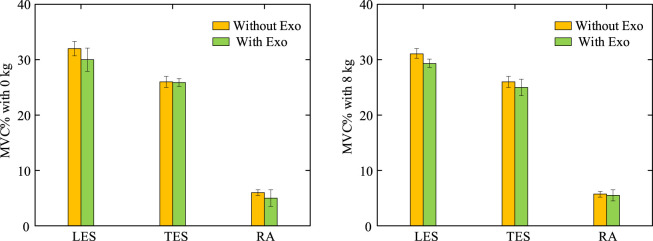
Percentage of peak EMG signals under dynamic bending and rising exercise. Comparative experiments with and without the exoskeleton were performed at 0 kg and 8 kg, confirming that the exoskeleton does not place an additional burden on the muscles.

The exoskeleton assist mechanism met the expectations of the objective function planning. At 8 kg weightbearing, muscle activation before and after wearing the exoskeleton exhibited the following changes (Figure 8): during the 0%–25%, 50% and 75%–100% phases of the flexion-extension exercise cycle, which are phases in which the lumbar spine is in mild flexion or deep flexion, the muscle activation had a similar trend with or without wearing the exoskeleton, consistent with the lack of active muscle force during the flexion-relaxation phenomenon. Since the traction force of the exoskeleton can also generate a partial extension moment, a trend of reduced muscle activation after wearing the exoskeleton was observed during the partial flexion-extension movement interval. Simulation results showed that the activation of the rectus abdominis muscle did not change much before and after the subjects wore the exoskeleton, and never exceeded the upper limit of 0.2. The experimental results (Figure 9) showed that, in the 0 kg experimental group, there was no significant difference in the level of EMG signals in the thoracic erector spinae muscle after wearing the exoskeleton (p = 0.62). The peak EMG signals of both the lumbar erector spinae and rectus abdominis muscles were slightly reduced, by 6.41% in the lumbar erector spinae (p = 0.036) and by 18.08% in the rectus abdominis (p = 0.02). In the 8 kg experimental group, the level of electromyographic signals in the thoracic erector spinae muscle after wearing the exoskeleton was also not significantly different (p = 0.226). Both the lumbar erector spinae and rectus abdominis muscles also had a slight decrease in peak EMG signals, with the lumbar erector spinae decreasing by 7.32% (p = 0.015) and the rectus abdominis decreasing by 16.03% (p = 0.023). The peak EMG signal of the rectus abdominis was consistently much lower than that of the erector spinae in both the 0 kg and 8 kg experiments and no significant elevation of the peak EMG signal of the rectus abdominis was observed with the exoskeleton. Because of the low extension moments generated, the results of both simulations and experiments observed a low impact of the exoskeleton on the muscles, which is consistent with the original design intent that the traction generated by the exoskeleton is more focused on reducing disc pressure than on reducing muscle activation.

## 5 Discussion

This study introduces a novel passive lower back support exoskeleton, which leverages the principles of leverage to provide two types of traction forces. By sacrificing part of the chest traction force that generates extension torque, the exoskeleton incorporates a lever mechanism to create back traction, achieving a lower support torque and a higher upper limit on traction force. A theoretical mechanics model of traction force supports this concept: as lumbar and hip flexion increases, intervertebral disc compression forces, along with both types of traction forces, also increase. Previous electromyographic studies have shown that passive exoskeleton-assisted extension moment reduces some active muscle forces, although this approach has a specific load threshold. Early designs of flexible exoskeletons or biomechanical garments have reduced muscle exertion to some extent, indirectly decreasing intervertebral disc loading. However, as the traction force ultimately acts on the spine, its effect on reducing disc pressure is limited. Some exoskeletons incorporate energy-storing components at the hip joint to support the torso, focusing on generating extension moment to reduce muscle force. However, this design overlooks the contribution of passive tissues, significantly limiting lumbar kinematics.

In practice, for light or zero-load bending and lifting movements, lumbar flexion rather than hip flexion is often the dominant motion pattern. Excessive extension moment generated by traditional exoskeletons can place an additional burden on the user. During trunk flexion-relaxation, the body ceases to engage active muscle forces, making moment demands on the exoskeleton more sensitive. If the exoskeleton’s assistive moment exceeds a certain threshold, the user may need to compensate by other means, such as activating abdominal muscles to generate counteracting flexion moment or modifying movement patterns toward a squatting posture. Previous studies on passive back exoskeletons reported increased abdominal muscle activity, with forces from the erector spinae and rectus abdominis aligning in the direction of disc compression. The impact of this additional activation pattern on spinal stability remains highly debated. Ultimately, these studies concluded that reduced electromyographic signals do not necessarily indicate reduced spinal load, and the relationship between the two remains unclear, with potential concerns about spinal loading (Koopman et al., 2020a; Koopman et al., 2019). In basic industry, lumbar compression force is a critical measure of spinal injury risk, as it can lead to disc herniation, one of the most common lumbar disorders (Jaffar Nur and Abdol, 2017). Traction forces, moreover, have been proven to effectively reduce disc compression, with therapeutic traction reaching up to 500 N to achieve significant results in lumbar traction therapy (Zaïri et al., 2021). Thus, in workplaces where bending and lifting with light or zero loads is required—such as long-duration lumbar flexion for surgeons or crop harvesting for agricultural workers—lower support moment combined with higher traction may offer better adaptability. For such occupations, exoskeletons focusing on traction rather than assistive moment could be more advantageous. This forms the core feature of our exoskeleton design: sacrificing some extension moment to achieve higher traction, offering a new design perspective on lumbar exoskeletons tailored to occupational needs.

The lower back exoskeleton previously proposed by Chaerim Moon (Moon et al., 2022) employed a cross 4-bar linkage mechanism. However, due to the intrinsic properties of this mechanism, the efficiency of spring force transmission was low, necessitating a high spring stiffness to achieve the allowable standard for traction force. Furthermore, the relationship between the generated traction force and moment in this exoskeleton was not well-defined. In contrast, the dual traction forces in our exoskeleton design have minimal pressure angle deviations from the direction of intervertebral disc pressure, resulting in notably enhanced force transmission performance.

Additionally, considering the ligaments, intervertebral discs, and passive muscle forces, we set an overall traction force limit of 500 N (as higher traction could be harmful to the lumbar spine) (Zaïri et al., 2021). Optimal control was used to determine the optimal stiffness of the exoskeleton springs, thereby defining the traction force range. The optimization objective function aligns not only with the body’s actual force generation patterns but also conforms to the original design of the exoskeleton to minimize the pressure on the L5/S1 disc. The simulation framework also provided predictions of intervertebral disc loading, which are difficult to measure in real experiments. The simulation results indicate that the optimized stiffness reduced the activation level of the erector spinae muscle group, and at maximum lumbar flexion, the peak compressive force on the intervertebral discs was effectively reduced.

The EMG shows that the optimized stiffness during the flexion and rise phases does not significantly increase abdominal muscle activity during the flexion and lifting phases, while activation of the thoracic longissimus and iliocostalis muscles decreased. This indicates that the extension moments generated by chest traction force is greater than the flexion moments generated by back traction force. This means that the extension moment produced by chest traction is greater than the flexion moment produced by back traction. The sum of the traction forces was also within the size range of effective traction forces and did not exceed the upper limit of more than 500 N. If the exoskeleton generated excessive extension moments, additional flexion moments are required to achieve moment equilibrium, and therefore, unintentional abdominal muscle activation may occur.

This study has several limitations. First, our prototype was designed as a rigid structure, with the assumption that the exoskeleton (made from lightweight materials) has zero mass, thereby neglecting the impact of inertial forces on the spring force and leverage. Second, our study was conducted solely in the sagittal plane, restricting lateral bending and rotation, and thus the effects of forces in other directions on intervertebral disc pressure remain unknown. Third, the connection between the exoskeleton and the human body was assumed to be fixed, but in reality, friction and damping coefficients between materials and the body may lead to relative slippage of the straps along the torso (Samper-Escudero et al., 2022), which could impact spring compression and the direction of traction force. Future research may need to establish a human-machine interaction model to predict the effects of such slippage, possibly by using materials with higher friction coefficients for the straps to minimize slippage. Fourth, the stiffness optimization was based on inverse dynamics; while this approach captures how stiffness affects muscle activation and disc pressure during standard movements, in reality, due to neurological reflexes, environmental factors, etc., exoskeletons may cause psychological or physiological burdens on the wearer and alter kinematics (Rimmele et al., 2023). In particular, the movement rhythm of the spine and spinal stability may change, which cannot be predicted by inverse dynamics. In the future, it is necessary to establish a forward dynamics model and make improvements based on the existing musculoskeletal models. For example, modify the description of spinal movement rhythm and evaluate movement stability through the position of the center of pressure (CoP) within the support polygon (Shan et al., 2025). This approach can predict more information in the absence of experimental data (Jin et al., 2024). Finally, the experimental subjects were limited to healthy young males and the experimental period was relatively short. If the diversity of subjects can be realized and the experimental period can be lengthened, it will be helpful to reveal the adaptability of exoskeletons to the general population and to consider muscle fatigue and ligament viscoelasticity in subjects wearing exoskeletons for a long period of time (Ojha et al., 2024).

## 6 Conclusion

In this study, we developed an exoskeleton that provides two types of traction forces using a single spring. The exoskeleton was designed for work environments involving light load lifting and bending, as well as addressing the compressive characteristics of spinal pressure during flexion relaxation. Its novelty lies in the use of a lever mechanism to sacrifice moment in exchange for additional traction force, resulting in lower moment support and higher traction force. Consequently, it demonstrates exceptional performance in reducing intervertebral disc pressure without compromising active muscle force generation. The traction force model estimates the trends of the two forces. Optimal traction force ranges were determined through stiffness optimization, and reductions in intervertebral disc pressure peaks and cumulative loads were estimated. Electromyography results indicate that wearing this exoskeleton reduces active muscle force, potentially alleviating lower back pain caused by prolonged or repetitive bending and lifting. Future work will focus on lightweight design considerations to ensure both wearer comfort and the mechanical reliability of the exoskeletons.

## Data Availability

The raw data supporting the conclusions of this article will be made available by the authors, without undue reservation.
